# Working Women and Maternity

**Published:** 1920-10-30

**Authors:** 


### THE PUBLIC WELL-BEING.

### Working Women and Maternity.

### MINISTRY OF HEALTH'S REPORT.

There will be other opportunities of dealing at more length with the extraordinarily full, complex and interesting annual report which has just been issued from the Ministry of Health by the Chief Medical Officer. As readers of the Public Well-being Section of the The Hospital are specially concerned in the question of maternity and child-welfare, we will content ourselves in this issue with some broad reference to the noteworthy chapter in the book which discusses this important matter from many points of view. Sir George Newman (the chief medical officer) points out that the problem is now for the first time supervised by one department of the State. There must come a time, he asserts, when civilisation means that no child-bearing woman is without adequate and skilled assistance, and no infant without a birth-right of health. "*That time should come in England first among the nations.*"

During the past twenty years of the present century there has been a remarkable fall in the total infant mortality rate, and recently also from diarrhœal diseases. "It is impossible to assess with exactness on the relative value of the numerous factors which have contributed to the remarkable decline in infant mortality, but it cannot, I think, be doubted that improved sanitation in its widest sense, and more particularly maternal and infant welfare work, has been a determining factor. The result cannot fail to bring with it a higher standard of national life and health."

Dr. Janet M. Campbell, after making a valuable and exhaustive analysis of problems involved in this branch of the report, comes to a number of general conclusions which it is worth while to quote. The ultimate objects of all effort for the welfare of mothers and little children, she says, should never be lost sight of—namely, to make maternity safer and less burdensome, less disabling for the mother; to give the mother a sound and practical knowledge of the elementary laws of hygiene which should govern the healthy upbringing of her family; to aid her to carry out these rules in her own home and to make good unavoidable shortcomings due to faulty environment, lack of means or social disability, by the provision of medical care in its widest connotations for the infant and young child both before and after birth.

Much interest has been displayed by working women themselves in all questions relating to maternity and child-welfare. They have suffered most in the past. They are becoming less disposed passively to accept the preventable ills which so often render the bearing and rearing of children an intolerable load and rob the function of motherhood of most of its happiness, and they are demanding an enlightened programme of reform. But although it is easy to enlist the co-operation of the intelligent working woman, it is extremely difficult to obtain a response from the less educated, slovenly woman whose standard of living, which never perhaps was good, has deteriorated as a result of bad surroundings. Yet she and her children are in urgent need of help if we can only persuade her to accept it.

Possibly working women themselves could reach the poorest mothers more effectively than has been possible hitherto. They can appreciate as no one else can the disastrous effects on health of poverty and its concomitant evils, and they have shown themselves fertile in suggestions for remedy. "It is eminently desirable that their services, together with those of women in other positions of life, should be utilised in an advisory and, when practicable, in an executive capacity in relation to all schemes for maternity and child welfare." The Ministry note with satisfaction in this connection that several local authorities have co-opted working women on to the maternity and child welfare committees.

The whole of this section of the report, of which the passages quoted above form the concluding part, deserves careful and sympathetic scrutiny.

